# Measuring human rights violations in a conflict-affected country: results from a nationwide cluster survey in Central African Republic

**DOI:** 10.1186/1752-1505-5-4

**Published:** 2011-03-07

**Authors:** Alina Potts, Kathleen Myer, Les Roberts

**Affiliations:** 1 Program on Forced Migration and Health, Heilbrunn Department of Population and Family Health, Mailman School of Public Health, Columbia University, 60 Haven Ave B-4, Suite 432, New York, NY, USA

## Abstract

**Background:**

Measuring human rights violations is particularly challenging during or after armed conflict. A recent nationwide survey in the Central African Republic produced estimates of rates of grave violations against children and adults affected by armed conflict, using an approach known as the "Neighborhood Method".

**Methods:**

In June and July, 2009, a random household survey was conducted based on population estimates from the 2003 national census. Clusters were assigned systematically proportional to population size. Respondents in randomly selected households were interviewed regarding incidents of killing, intentional injury, recruitment into armed groups, abduction, sexual abuse and rape between January 1, 2008 and the date of interview, occurring in their homes' and those of their three closest neighbors.

**Results:**

Sixty of the selected 69 clusters were surveyed. In total, 599 women were interviewed about events in 2,370 households representing 13,669 persons. Estimates of annual rates of each violation occurring per 1000 people in each of two strata are provided for children between the ages of five and 17, adults 18 years of age and older and the entire population five years and older, along with a combined and weighted national rate. The national rates for children age five to 17 were estimated to be 0.98/1000/year (95% CI: 0.18 - 1.78) for recruitment, 2.56/1000/year (95% CI: 1.50 - 3.62) for abduction, 1.13/1000/year (95% CI: 0.33 - 1.93) for intentional injury, 10.72/1000 girls/year (95% CI: 7.40 - 14.04) for rape, and 4.80/1000 girls/year (95% CI: 2.61 - 6.00) for sexual abuse. No reports of any violation against a person under the age of five were recorded and there were no reports of rape or sexual abuse of males. No children were reported to have been killed during the recall period. Rape and abduction were the most frequently reported events.

**Conclusions:**

The population-based figures greatly augment existing information on human rights violations in CAR, and represent a step forward in quantifying the protection needs of Central Africans. Government, donors, and international organizations should make use of this data to better inform advocacy, prevention, and response programs, to assist in fundraising, and to develop surveillance activities to monitor child protection concerns.

## Background

Measuring human rights violations is difficult even in the best of circumstances, and particularly challenging during or after conflict, when populations are under extreme duress and the systems needed to support them have been disrupted or destroyed [1,2]. While data derived from surveys or surveillance systems is crucial to informing the funding and planning necessary for an effective humanitarian and public health response, these systems often do not track violations of individuals' rights. Where documentation of human rights abuses in conflict is available, it is often based on individual case reports, which provide detailed descriptions but are likely to represent only a small portion of actual events [3].

To gain a more complete understanding of the extent and nature of human rights violations, case reports and qualitative situational analyses should be augmented with population-based estimates that provide data on the scope and magnitude of the problem [4]. In taking this mixed methods approach, qualitative reports that provide information about the experience of those affected and the context in which violations occur are complemented by quantitative methods, to determine approximately how many people are affected, which violations are most prevalent, with what frequency they occur, and where. Several rigorous, quantitative studies of the prevalence of human rights abuses and sexual violence in conflict have been undertaken in recent years [1,3,5-8], and epidemiological methods [9] have increasingly been used to explore rights violations. Yet such studies remain rare and lack uniform methods. Thus, there exists a need to develop appropriate methods to capture these data in insecure, logistically challenging, and culturally and politically sensitive environments.

The United Nations (UN) has increasingly asked country programs in conflict areas to collect such data and to specifically address the care and protection of children. UN Security Council Resolution 1612 requested the immediate establishment of a monitoring and reporting mechanism (MRM) "to collect and provide timely, objective, accurate and reliable information" on "grave" violations against children affected by armed conflict [10]. The MRM seeks to monitor the following six grave violations against children: killing and maiming; abduction; attacks against schools and hospitals; rape and sexual violence; denial of humanitarian access; and recruitment or use of child soldiers (now referred to as children recruited or used by armed forces or armed groups) [11]. While MRM processes differ among countries, they currently rely on "verified" cases. This refers to a passive surveillance system that relies on incident reports captured by NGO partners implementing programs, or by community-based monitoring led by civil society organizations. These incidents are then confirmed, normally by a UN agency, through other sources. The collection of population-based data would complement this process, and provide the UN and other actors accountable for the protection of children with broader estimates of both the scale and spread of rights violations in conflict. In December 2007, Columbia University, the U.S. Centers for Disease Control and Prevention, and UNICEF initiated collaboration on developing population-based methods to capture grave violations capable of complementing the current practice of case-based MRM monitoring.

Conflict in the Central African Republic (CAR) is one of 20 situations of concern in the UN Secretary-General's 2009 report to the Security Council on children and armed conflict [12]. One of the world's poorest and least healthy states, CAR ranks 179^th ^out of 182 countries in the 2009 Human Development Index rankings [13]. Multiple armed actors are active in CAR, including relatively weak government armed forces, five formal and recognized non-state armed groups, and various other non-state armed elements, such as village self-defense groups and road bandits-known locally as Zaraguinas-who operate throughout the country with impunity [14]. Due to the meager humanitarian presence outside the capital city, little information is available on the human (or child) rights abuses occurring or on the full extent of the health and security crisis facing the country. Thus, in June and July of 2009, a Columbia University research team, with the support of the United Nations Children's Fund (UNICEF) in CAR, executed a national, stratified, four-stage cluster survey to obtain estimates of the rates of four of the grave violations committed against children: killing or maiming, abduction, recruitment, and rape or sexual abuse. Attacks against schools or hospitals and denial of humanitarian access for children were not included at this time, as they occur at the community level rather than at the individual or household level at which the survey was conducted.

## Methods

In the 1980s, the "sisterhood method" was developed as an efficient means of measuring maternal mortality through population-based surveys. With this method, adult respondents are asked questions about the survival of siblings sharing the same mother [15]. Since maternal deaths are a relatively rare phenomenon, the ability to capture information on multiple persons through one respondent expands the effective sample available for analysis given a fixed number of data collection contact points, making data collection more efficient and avoiding the prohibitive costs associated with obtaining very large sample sizes. Building on this and subsequent revisions, Columbia University developed the "Neighborhood Method" to measure sensitive events, such as rape and sexual abuse, in situations of humanitarian concern where security, logistical and financial constraints can make large samples difficult to obtain.

In this method, interviewers conduct one-on-one, in-depth, household-based interviews, asking about respondents' experiences as well as the experiences of all members of their household, and members of the households of their three closest neighbors. The neighborhood method reproduces the efficiency of the sisterhood method, while also allowing two lenses through which respondents can speak about sensitive topics: disclosure of their and their family's experiences, as well as anonymous disclosure of their neighbors' experiences. These lenses also offer potential sources of validation, such as the potential to examine under-reporting by interviewees of their own adverse experiences [16].

The survey in Central African Republic represented the fifth time the neighborhood method has been executed in a conflict or post-conflict setting, following pilots in northern Uganda (2006) [17] with Christian Children's Fund, in Liberia (2007) [2] and Ethiopia's Somali region (2008) [18] with the International Rescue Committee, and in Sri Lanka (2008) [16] with Save the Children.

### Protocol and Ethical Considerations

The research protocol was developed building on published guidelines for working with sexual violence survivors [19], as well as operational guidelines utilized by members of the research team during previous work in gender-based violence response. First and foremost, consideration was given to the sensitive topics to be covered and possible negative repercussions arising from such discussions, for both respondents and interviewers. Interviews took place only with women 18 years of age or older, in a private space, and after obtaining informed consent and notifying the respondent that she could end the interview at any time. Information on health and social services available in the different areas of CAR was obtained to enable interviewers to refer respondents to services if needed; however, it should be noted that access to services in most areas of CAR is severely hampered due to insecurity, lack of public or private means of transport, and poor quality roads. During field research, if the survey team came across a person deemed to be in dire need of immediate medical assistance, the team would provide her or him with transportation to the nearest health facility.

Confidentiality of neighbor reports was enhanced by a revision to the method in which small objects were used to represent the aggregate number of individuals residing in neighboring households (see further discussion below). In order to monitor the safety of survey respondents, households in some villages were re-visited several days to a week after the initial interview, and respondents were asked about any positive or negative consequences following their interview. If a negative incident was reported, the survey team would be able to gain insight into the possibility of risk to future respondents. If more than one violent incident were reported, the survey would come to an immediate halt. Columbia University's Institutional Review Board approved the protocol.

### Interviewer Training and Questionnaire

The survey team consisted of three researchers from Columbia University and six Central African interviewers. Training of the interviewers took place over five days. Most interviewers had past survey experience with non-governmental organizations or private companies. All were female, university-educated, and fluent in French and Sangho, a language spoken nationally. Many interviewers spoke other local languages as well. The training method included role play, didactic sessions, and practice interviews. Training began with an open discussion on child protection and gender-based violence issues in CAR, followed by extensive discussions on the meaning and definitions of the grave violations being measured. Other topics covered included working with survivors of violence, informed consent and maintaining confidentiality, surveying methods and data collection techniques, and referral protocols. One of the interviewers, a trained social worker, co-facilitated the session on gender-based violence. The questionnaire and data collection forms were revised throughout the training, and field-tested during two days of pilot interviews conducted in Bangui.

The questionnaire was based on those used in previous iterations of the method. Interviewer input assisted in placing each definition within a local context and wording questions such that local populations would best understand what was being asked of them. For example, maiming was defined locally using a term translatable in English as injury, and defined so as to include incidents of killing. The definitions for each grave violation, as agreed upon by the research team, are as follows:

• **Injury**: Any type of injury to a person, accidental or intentional, including killing and other injuries that resulted in physical harm. Only non-fatal injuries recorded as intentional and non-self-inflicted were counted as an injury violation. Intentional, non-self-inflicted injuries resulting in death were counted as a killing violation.

• **Recruitment**: Being approached by or recruited to join armed groups, forcibly or otherwise (i.e. voluntarily).

• **Abduction**: Being forcibly taken or disappearing from a home or village.

• **Sexual Abuse**: Unwanted verbal harassment or attempts at touching; unwanted sexual advances such as kissing, groping, etc.; or attempted rape, defined as an attempt at non-consensual penetration of an orifice by an object or the body part of another person, without the actual act of penetration taking place.

• **Rape**: Penetration of an orifice by an object or the body part of another person, without one's consent.

Questionnaires were written in French, and a Sangho version was verbally agreed-upon by the interview team.

### Sampling

**Figure 1:**
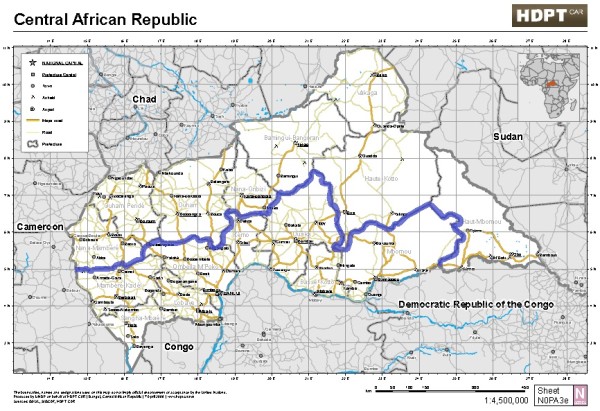
**Map of the Central African Republic showing the delineation of the northern and southern strata**.

Sampling was based on 2003 census data from the *3ème Recensement Général de la Population et de l'Habitation de 2003 (RGPH03)*, obtained from the UN Office for the Coordination of Humanitarian Affairs (OCHA) in Bangui. Previous surveys in CAR [20] and the experience of colleagues indicated that there is generally better economic status and development indicators in Bangui and the southern areas of the country, and identified the majority of recent or current conflict zones lying in the northern half of the country, which is close to neighboring conflicts in Chad and Sudan. To increase the survey's precision, the country was divided into two strata based on these differences (see figure 1). The "northern stratum" included Bamingui Bangoran, Nana Gribizi, Ouham, Ouham Pendé, Nana Mambéré, Haut Kotto, Haut Mbomou, and Vakaga prefectures. This area has an estimated population of 1,394 million people. Prefectures considered to be less affected by conflict and within closer reach of government services were Mambéré Kadéï, Sangha Mbaéré, Lobaye, Ombella M'Poko, Bangui, Kémo, Ouaka, Basse Kotto, and Mbomou. This area is referred to as the "southern stratum" and has an estimated population of 2,501 million people.

A minimum effective sample size of 864 individuals (providing data on at least 6 female neighbors) in each stratum was required in order to estimate with 95% confidence the proportion experiencing rape to within 2% and assuming a true prevalence of 10% (calculated using Epi Info, Version 6, CDC Atlanta, 1993). A design effect of two was assumed, suggesting a sample of 300 households (30 clusters of 10 households) per stratum. A household was defined as people who eat together, and for the purposes of enumeration of the sample, included all members who had slept there the previous night.

Security and logistical constraints prevented the team from planning data collection activities in Vakaga and Haut Mbomou prefectures. Because of this, and the assumption that further logistical constraints and UN security regulations would arise during data collection, 36 clusters were selected from the northern stratum and 33 were selected from the southern stratum, with a goal of obtaining 30 clusters from each. Cluster selection was performed in four stages by sampling population proportional to size (PPS): first selecting the number of clusters per prefecture, followed by the number of clusters per commune (administrative district) within the prefecture, then the specific village or urban neighborhood, and finally the initial household. It was determined that by including the no-travel areas in the sampling frame, and then adding those missed clusters to the villages that were unreachable, the team could best articulate the fraction of the population missed because of security and logistical concerns. If, during surveying, a selected cluster was inaccessible not because of security but due to a damaged bridge, flooding, or some other logistical constraint, the nearest accessible town or village to that selected cluster was used as a substitute.

Within each selected cluster (either a village or urban neighborhood), permission to work was first sought from the locally identified leader, usually the village chief. The chief or a representative would accompany the research team to ascertain the village's or neighborhood's borders and create a rough map of the cluster. In small clusters, the team was able to count the total number of households, while in more populated clusters, the total was estimated by breaking the cluster into geographically distinct sections, counting the households along two axes of each section, and multiplying these together as an estimate of household density. The sampling interval was determined by dividing the total number of households by 10. The smallest interval used, regardless of the number of households in a given location, was six, guaranteeing that a selected household's neighbors would not then be re-selected as a household in the sample. If a village consisted of fewer than 60 households, interviewing would continue in the same direction, using the same sampling interval of six, into the nearest adjacent village. Because of the need to complete interviews and return to UN-approved overnight locations before curfew, the largest interval used was 20.

The first household to be interviewed was selected at random. In large villages and urban areas, this process sometimes occurred in two parts. First, the rough map of the cluster's layout and density was used to divide it in half, or for very large clusters, 3-5 distinct sections (of no more than 200 households each), and a coin flip or random digit generator (paper currency) was used to select the section in which surveying would begin. A random number between one and the sampling interval would then be selected from the numbers (beginning on the right) of a different piece of paper currency. The next nine households were selected systematically in an attempt to have a sample that was spread evenly over the village or neighborhood. The ten households to be interviewed, and the three nearest neighbors of each, were identified by the team leader before interviewers approached the household.

### Data Collection

On approaching the household, interviewers introduced themselves as members of a UNICEF team interested in learning about the situation of women and children, and asked to speak with the female head of household. After explaining the purpose of the visit and ensuring that the woman was at least 18 years of age, the interviewer would invite her to participate in an interview and ask to move to a private space in or near the home. The interviewer would then read an informed consent statement and record the respondent's oral consent and her age and marital status on the data collection form. If no eligible respondents were home or willing to participate, the interviewer would go to the next nearest neighbor of the three households previously identified by the team leader. If the interviewer encountered four refusals or unavailable households in a row, she would then proceed to the household at the start of the next sampling interval. In one cluster where many such "skips" occurred due to most of the village being away, several homes were revisited and the interviews conducted with those absent during the original day of data collection. This was done to determine the potential for absentee bias associated with the initial skips, and data from these revisits are not included in the analysis.

Interviewers were encouraged to collect information via semi-structured conversations rather than by a series of rigid questions. Questions regarding household composition, births, and deaths since January 1, 2009, were followed by a more general probe of the problems facing women and children in the respondent's village or neighborhood. In many cases, this would lead to a respondent-initiated discussion related to one or more of the violations under study, and the interview could be built on these initial concerns. The interviewer moved on to questions regarding the occurrence of each of the four grave violations since January 1, 2008; the longer recall period allowed for inclusion of events occurring before peace accords were signed in June and December 2008. At the end of the interview, women were asked what they thought would be the most helpful solutions to the problems discussed. If health or other services were available nearby, the interviewer would offer information on relevant services. Interviews typically lasted about 40 minutes.

Following the neighborhood method, all interview questions were asked both for the respondents' household and for those of her three closest neighbors. To facilitate this process, objects were used to represent the compositions of the three neighbor households. Different-sized bells or buttons of two different colors were used to denote both males and females above 18 years, males and females between 5 and 17 years, and children below 5 years of age. As each additional household was described, more objects were added to the pile that represented the combined composition of the three nearest households; grouping the objects in this way added a degree of anonymity to the neighbor's information, in that the specific neighboring household of someone experiencing an incident was not indicated to the interviewer. When events were discussed, interviewers could refer to the objects to ensure that the person experiencing the incident was one of the neighbors previously described. In the case of a death, an object could be placed in the collection to confirm that there was previously another person living among this group. If an interviewee did not know a household well enough to provide at least its crude composition, the interviewee was not asked about that household.

### Analysis

In order to estimate the annual rate of each incident and provide a breakdown of these rates by age, each member of the sample population was analyzed individually, regardless of her or his status as belonging to a respondent ("self-report") household or a neighboring household. If an individual experienced multiple violations, for example rape and abduction, this would be counted as both a rape and an abduction. However, if the same individual survived repeat incidents of the same violation (i.e. multiple rapes), the first instance of the given violation occurring within the recall period is the only one counted.

Data were analyzed by strata. Annual incident rates and confidence intervals were calculated with software designed for Save the Children by Mark Myatt (Institute of Ophthalmology, UCL, London, UK), which takes into account the design effect associated with cluster surveys. Nationwide estimates were created using WINPEPI Describe version 1.44 (J.H. Abramson, 2004), which weighted strata proportional to their 2003 census population in making point estimates and confidence intervals. For confidence interval calculations, the entire sampling universe was assumed to have the same age and gender breakdown as the sample taken (for example, 16.7% of the sample in the northern stratum is <5 years old, so the entire population of <5-year-olds in the northern stratum was assumed to be 16.7% of the 1,394 million people comprising the entire 2003 census estimate for that area). The midpoint date of the interviewing process was used for a recall period of 18.1 months. A steady-state population, where the number of people entering the age category was equal to the number of people exiting over the recall period, was assumed as the denominator for each rate. Within-sample descriptive statistics were calculated with SPSS 16.0 (SPSS Inc, 2007).

## Results

In total, 599 women were interviewed about events in approximately 2,370 households across the two strata: 310 women in the southern stratum and 289 women in the northern stratum. The vast majority of women interviewed were able to report on the situations of the residents in all three neighboring households, with only 17 of the 599 respondents unable to do so. The population living in respondent households ("self-report households") and their closest neighboring households ("neighbor-report households") represented in the survey is 13,669 persons: 6,668 in the northern stratum and 7,001 in the southern stratum (Table 1). The median age of respondents was 28 years (range 18-80) and the majority (70%) was married, with 14% single, 12% widowed, and 4% divorced.

**Table 1:** Sample Population by Age, Gender, and Strata: Self-Report and Neighbor-Report Household (HHs)*

	Under 5		5-17				18+				TOTAL
		
	*Self-Report HHs*	*Neighbor-Report HHs*	*Self-Report HHs*		*Neighbor-Report HHs*		*Self-Report HHs*		*Neighbor-Report HHs*		
		
			Male	Female	Male	Female	Male	Female	Male	Female	
**North**	367	795	337	359	852.5	912	353	469	986.5	1236.5	**6668**

**South**	384	861	349	375	854	949.5	432.5	564	1030	1201.5	**7001**

	751	1656	686	734	1706.5	1861.5	785.5	1033	2016.5	2438	**13669**

* Non-integer numbers arose as the result of partial person-time being added for household members reported to have experienced one of the grave violations that resulted in their removal from the household at some point during the recall period. These numbers were always rounded to the nearest integer when totaled.

Of the 69 villages or urban neighborhoods selected into the sample as clusters, 9 (7 in the north, 2 in the south) were inaccessible and could not be substituted with a nearby village or neighborhood. Of these, 3 were located in prefectures partly or wholly excluded from the survey due to both security and logistical constraints (Vakaga, Haut Mbomou, and Bamingui Bangoran). The total population represented by these missed clusters was approximately 19% in the north (7/36) and 6% in the south (2/33). On 9 other occasions (4 in the north, 5 in the south), the specific village selected could not be reached because of a logistical barrier, but a neighboring village was selected as a substitute.

Seven women (1.2%; 2 in the south, 5 in the north) refused to be interviewed for varying reasons. Two of the refusals occurred in a set of households where no neighbors were present to be interviewed either. No response was obtained on 63 occasions (9.5%), when the selected household and all 3 neighbors were not at home. On a separate 285 occasions (32%), no eligible respondent was home at selected households, but one of the other three neighbors was successfully interviewed about the same group of 4 households.

### Self vs. Neighbor Reports

Information for each grave violation, broken down by respondents' reports of incidents in their own households and those occurring in their neighbors' households, are reported in Table 2. The annual rates (combined & weighted national estimates) of injury and rape were about twice as high among self-report households as among neighbor-report households. For abduction and sexual abuse, the rates reported among self-report and neighbor-report households were similar. Comparison between reports of killing, and of recruitment, is not possible due to the low number of incidents reported. By examining the confidence intervals surrounding the directly estimated difference in prevalence between self-report and neighbor-report households (among both strata combined), we noted no statistically significant difference between the occurrence of self-reported events and those reported to have occurred in neighboring households, except for reports of rape and intentional injury. Statistically significant differences (i.e. 80% confidence intervals do not overlap) between self-reported and neighbor-reported incidents exist for the reporting of rape in the northern and southern strata as well as the combined national estimate, and for the reporting of intentional injuries in the combined national estimate.

**Table 2:** Reported Violations Among Self-Report vs. Neighbor-Report Households (HHs)

		North		South		Combined & Weighted National Estimate	
		
		*Incidents Recorded*	*Annual Rate/1000 people (95% CI)*	*Incidents Recorded*	*Annual Rate/1000 people (95% CI)*	*Incidents Recorded*	*Annual Rate/1000 people* *(95% CI)*
Recruitment	Self-Report HHs	1	0.44 (0 - 1.35)	0	N/A	1	0.16 (0 - 0.49)
	
	*Neighbor-Report HHs*	17	2.83 (0.53 - 5.13)	0	N/A	17	1.02 (0.19 - 1.84)

Abduction	Self-Report HHs	26	11.36 (6.78 - 15.93)	1	0.39 (0 - 1.21)	27	4.32 (2.60 - 6.05)
	
	*Neighbor-Report HHs*	90	15.00 (10.19 - 19.81)	5	0.82 (0.03 - 1.61)	95	5.91 (4.11 - 7.71)

Rape	Self-Report HHs	41	32.83 (21.03 - 44.63)	32	22.59 (13.27 - 31.92)	73	26.29 (18.96 - 33.61)
	
	*Neighbor-Report HHs*	76	23.52 (17.16 - 29.88)	32	9.86 (6.84 - 12.89)	108	14.79 (11.79 - 17.79)

Sexual Abuse	Self-Report HHs	9	7.21 (3.14 - 11.27)	3	2.12 (0 - 4.57)	12	3.95 (1.81 - 6.10)
	
	*Neighbor-Report HHs*	30	9.28 (5.96 - 12.61)	8	2.47 (0.47 - 4.47)	38	4.92 (3.17 - 6.68)

Killing*	Self-Report HHs	4	1.75 (0.18 - 3.32)	0	N/A	4	0.63 (0.06 - 1.19)
	
	*Neighbor-Report HHs*	13	2.16 (0.99 - 3.34)	1	0.16 (0 - 0.47)	14	0.88 (0.42 - 1.35)

Intentional Injury*	Self-Report HHs	22	9.61 (4.89 - 14.33)	12	4.62 (2.29 - 6.96)	34	6.41 (4.15 - 8.67)
	
	*Neighbor-Report HHs*	30	4.99 (2.26 - 7.71)	12	1.97 (0.47 - 3.47)	42	3.06 (1.68 - 4.43)

* Killing and intentional injury are mutually exclusive categories.

### Point Estimates

No reports of any violation against a child under the age of five were recorded and there were no reports of rape or sexual abuse of males. For this reason, all point estimates (Table 3) were calculated using the sample population five years of age and over, and using only females when calculating estimates of rape and sexual abuse.

**Table 3:** Reported Violations by Age Group and Strata

	North		South		Total Incidents Recorded	Combined & Weighted National Estimate	
			
	Incidents Recorded	Annual Rate/1000 People (95% CI)	Incidents Recorded	Annual Rate/1000 People (95% CI)		Annual Number of Incidents (95% CI)	Annual Rate/1000 People (95% CI)
Violation	**Children 5-17**						

Recruitment	10	2.70 (0.50 - 4.89)	0	N/A	10	1392 (252 -2520)	0.98 (0.18 - 1.78)

Abduction	21	5.67 (3.15 - 8.18)	3	0.79 (0 - 1.64)	24	3624 (2124 - 5136)	2.56 (1.50 - 3.62)

Rape	32	16.72 (10.44 - 23.00)	15	7.51 (3.67 - 11.34)	47	8172 (5640 - 10704)	10.72 (7.40 - 14.04)

Sexual Abuse	21	10.97 (5.96 - 15.99)	3	1.50 (0 - 3.53)	24	3660 (1992 - 5340)	4.80 (2.61 - 6.0)

Killing*	0	N/A	0	N/A	0	N/A	N/A

Intentional Injury*	3	0.81 (0 - 1.69)	5	1.31 (0.16 - 2.46)	8	1596 (468 - 2736)	1.13 (0.33 - 1.93)

	**Adults >= 18**						

Recruitment	8	1.74 (0 - 3.55)	0	N/A	8	1104 (0 - 2256)	0.62 (0 - 1.26)

Abduction	95	20.73 (13.94 - 27.51)	3	0.62 (0 - 1.32)	98	13908 (9516 - 18312)	7.77 (5.31 - 10.23)

Rape	85	33.12 (25.01 - 41.24)	49	18.40 (12.44 - 24.37)	134	23414 (18672 - 28164)	23.72 (18.91 - 28.53)

Sexual Abuse	18	7.01 (4.38 - 9.65)	8	3.00 (0.13 - 5.87)	26	4392 (2352 - 6432)	4.45 (2.39 - 6.52)

Killing*	17	3.70 (1.93 - 5.48)	1	0.21 (0 - 0.58)	18	2592 (1380 - 3804)	1.45 (0.77 - 2.13)

Intentional Injury*	49	10.67 (6.35 - 14.99)	19	3.90 (1.40 - 6.41)	68	11292 (7308 - 15288)	6.31 (4.08 - 8.54)

	**Total Population >= 5**						

Recruitment	18	2.17 (0.46 - 3.88)	0	N/A	18	2496 (528-4464)	0.78 (0.16 - 1.39)

Abduction	116	13.99 (9.71 - 18.27)	6	0.69 (0.10 - 1.28)	122	17532 (12456 - 22608)	5.47 (3.88 - 7.05)

Rape	117	26.11 (20.13 - 32.09)	64	13.73 (10.46 - 17.00)	181	31416 (26220 - 36660)	18.20 (15.19 - 21.20)

Sexual Abuse	39	8.70 (5.86 - 11.55)	11	2.36 (0.69 - 4.03)	50	8028 (5472 - 10584)	4.65 (3.17 - 6.13)

Killing*	17	2.05 (1.07 - 3.03)	1	0.12 (0 - 0.33)	18	2592 (1380 - 3804)	0.81 (0.43-1.19)

Intentional Injury*	52	6.26 (3.72-8.81)	24	2.77 (1.34 - 4.19)	76	12900 (8748 - 17040)	4.02 (2.73 - 5.31)

* Killing and intentional injury are mutually exclusive categories.

Looking within the sample at the confidence intervals around reported incidents among all persons over 5 years old, those living in the north consistently experienced violations at a rate significantly higher than those living in the south.

### Recruitment

Eighteen incidents of forcible recruitment were reported, all in the northern stratum. Approximately 2.17 (95% CI: 0.46 - 3.88) people per 1000 in the north were forcibly recruited into armed groups each year over the recall period. It should be noted that only adults forcibly recruited by armed groups were included, while reports of both forcible and "voluntary" child recruitment were included. Both armed groups and Zaraguinas were responsible for recruitments. Of the 8 incidents among adults, 7 were males, with a median age of 21.5 years (range 18-32). All incidents were perpetrated by armed groups, except one man who was recruited by Zaraguinas. The median age among the 10 children recruited was 16 years (range 12-17). Six of the children were male and four were female. All incidents of child recruitment were perpetrated by armed groups, with the exception of two boys who were recruited as Zaraguinas. Two of the 16-year-old boys reportedly joined the armed groups "voluntarily". All incidents among females (1 adult, 4 children) involved an armed group first raping and abducting the victims.

### Abduction

Abduction was one of the most frequently reported violations, with 122 incidents reported. The vast majority (95.1%, n = 116) of these occurred in the north, where the estimated annual rate of abduction during the recall period was 5.67 per 1000 children ages 5 to 17 years (95% CI: 3.15-8.18), and 20.73 per 1000 adults over 18 years (95% CI: 13.94-27.51). Most abductions (64.8%, n = 79) occurred among adult men. Overall, perpetrators included armed groups (70, all in the north), Zaraguinas (44, of which 40 were in the north), unknown actors (5), and government forces (3). Among 19 adult women, 10 were taken by armed groups and 8 by Zaraguinas; in 6 cases rape by the perpetrator(s) was also reported, and in 1 case in the north a 50-year-old woman was also killed by her captors.

The 24 children affected ranged in age from 8-17 years (median = 15); 21 lived in the north and two-thirds of all abducted children were male (n = 16). The largest perpetrator of incidents against children remained armed groups (12) and Zaraguinas (11), with Zaraguinas taking only boys and armed groups abducting both boys and girls. Among the 8 reported abductions of girls, 7 were taken by armed groups, of whom 6 were also raped. The parents of one girl paid for her release and 5 were still missing at the time of interview. Within abductions perpetrated by government forces, 2 adult males taken by gendarmes were later killed, and another adult male taken by the Central African army was released following payment by the family. Over half (56.1%, n = 64) of the 114 abductions perpetrated by armed groups or Zaraguinas ended in the return of the person abducted, after a period ranging from several days to 3 months. Of these returns, 27 (42%) occurred after payment of ransom by a family member.

Table 4 illustrates a trend that emerged from the data, of abducting people for periods of time varying from a couple of days to three months, and using them for labor or holding them for ransom. Men were frequently abducted to help carry goods pillaged from a village during a raid by armed groups or Zaraguinas, and then returned several weeks later. Three respondents noted exact ransom amounts up to $300-$400 USD, sums far beyond the reach of most Central Africans.

**Table 4:** Abductees, Returnees and Ransoms by Age Group, Northern Strata

	Children 5-17		Adults >= 18		TOTAL
	
	male	female	male	female	
**Abducted**	16	8	79	19	**122**

**Returned (*Overall*)**	13	1	40	10	**64**

**Returned (*Ransom*)**	6	1	16	4	**27**

### Rape

Rape was the most frequently recorded incident within the sample, with 181 reports among women and girls (117 in the north, 64 in the south), allowing for a national estimate of 31,416 (95% CI: 26,220-36,660) rapes occurring each year during the recall period. The majority (74%, n = 134) were reported among adult women, ranging in age from 18 to 67 years (median = 22). The estimated annual rate of rape was 33.12 per 1000 adult women in the north (95% CI: 25.01-41.24) and 18.4 per 1000 adult women in the south (95% CI: 12.44-24.37). However, rape was also recorded among very young children: 47 rapes were reported against children ranging in age from 8 to 17 years old (median = 15). In the north, the estimated annual rate of rape was found to be 16.72 (95% CI: 10.44-23.0) per 1000 girls age 5 to 17. The rate in the south was less than half that found in the north (7.51 per 1000 girls per year, 95% CI: 3.67-11.34). Reported incidents allow us to estimate that 8,172 girls (95% CI: 5,640-10,704) across the country were raped in each year of the recall period.

Although the interviewer did not explicitly ask about marital rape, 80 incidents (44%) of women being raped by their husbands were reported, all among adult women except 2 incidents among married, 17-year-old girls. (While rape is illegal in CAR, the law does not specifically prohibit marital rape.[21]) Another 32 incidents were reportedly perpetrated by armed groups (17 among children), 26 by neighbors (16 among children), 16 by strangers (3 among children), 14 by Zaraguinas (2 among children), 5 by family members (4 among children), 4 by merchants (1 among children), 3 by "other" actors (including a 23-year-old raped by 5 people in a criminal gang, a 22-year-old raped by a man and later forced to marry him, and a 16-year-old raped by a 45-year old man in order to force her to marry him), and a 21-year-old female reportedly raped by a government soldier. With the exception of two incidents (1 by an armed group and 1 by Zaraguinas), the 25% of rapes perpetrated by armed groups or Zaraguinas (44) all occurred in the north.

Among all non-marital rapes (101), respondents reported that 9 resulted in pregnancy, and 4 occurred while the victim was pregnant. Four survivors were reported as sick following the incident, and a further 8 were said to be in the hospital. Eight gang rapes were reported, half perpetrated by armed groups and half by neighbors, usually in groups of 3-5 males. In two cases, the respondent noted that the survivor had been raped on multiple occasions. Four incidents resulted in talking to the chief, 2 in going to the police and 5 in the perpetrator's arrest. Forced marriage to the rapist was reported in 6 cases, and in 1 case the rapist paid the survivor's family 40,000 CFA but "refused" to marry her. Among rapes by family members, 2 were by the uncle, 1 by the aunt's husband's older brother, one in the aunt's household, and 1 reported as repeated forced sex by the husband's younger brother following the husband's death.

One rape also resulted in an intentional injury and another rape resulted in a killing. With 11 rapes (7 perpetrated by armed groups and 4 by Zaraguinas) the women or girls were also abducted, and among them 5 were also recruited into armed groups.

### Sexual Abuse

The sample included 50 reports of sexual abuse (39 in the north, 11 in the south) against women and girls, of which 24 were among children. In the north, a higher annual rate was seen among girls, with an estimated 10.97 (95% CI: 5.96-15.99) instances of sexual abuse per 1000 girls each year and an estimated 7.01 (95% CI: 4.38-9.65) per 1000 women. However, the opposite was found in the south, with the rate among women (3.00 per 1000/year, 95% CI: 0.13-5.87) twice that as the rate among girls.

Overall, 24 incidents (11 among girls) were classified as sexual harassment, 17 (7 among girls) as sexual abuse, 8 (5 among girls) as attempted rape, and 1 unknown (girl). Almost half of the incidents (42%, n = 21 including 10 among children) were perpetrated by neighbors. There were 11 incidents perpetrated by armed groups (8 among children), 6 by strangers (2 among children), 5 by husbands, 2 by other family members (1 among children), 2 by Zaraguinas (1 among children), 2 by merchants (both against children), and 1 by an additional perpetrator classified as "other." The ages of affected children ranged from 11-17 years (median = 15) and, for adults, the range was 18-55 years (median = 20).

All incidents perpetrated by members of armed groups or Zaraguinas occurred in the north, while only 1 incident perpetrated by a spouse was recorded against a 55-year-old female. Conversely, all incidents reported in the south were perpetrated by either husbands (36%, n = 4) or neighbors (64%, n = 7); all incidents perpetrated by a husband were against women over the age of 18. Among incidents of attempted rape, perpetrators included a husband (1 adult), a stranger (1 adult), armed groups (3, all children), Zaraguinas (1 adult, 1 child) and a neighbor (1 child).

### Killing

Eighteen reports of violent deaths were recorded within the sample during the 18.1 month recall period, all among adults and all but 1 in the northern stratum. The ages of those killed ranged from 18-62 years (median = 34). Nearly half of perpetrators were armed groups (44.4%, n = 8), including one report of a man killed after being convicted of sorcery. Four killings were reportedly perpetrated by Zaraguinas, 3 by government forces (gendarmes or members of the military), 2 by other or unknown persons, and 1 by a stranger. Of those killed, 3 were females, including 2 women in the north killed by armed groups (ages 38 and 50 years, one of whom had been abducted) and 1 woman in the south (age 25 years) who was raped and then strangled to death by a stranger. Incidents recorded within the sample allow for an estimate of 2,592 (95% CI: 1,380-3,804) adults killed each year over the course of the recall period.

### Intentional Injury

The survey captured 76 incidents of intentional injury, of which approximately two-thirds (68.4%, n = 52) occurred in the north. There, an estimated 6.26 per 1000 people per year (95% CI: 3.72-8.81) were intentionally injured over the course of the recall period. The rate is much lower in the south, at 2.77 per 1000 people per year (95% CI: 1.34-4.19).

Within both strata, the vast majority of incidents occurred among adults (89.5%, n = 68), and ages ranged from 18 to 57 years (median = 27.5). Among those injured in the north, over half (57.7%) were male while the opposite was true in the south, where 58.3% (n = 14) were female. The top 3 perpetrators were armed groups (n = 22), spouses (n = 17, all but one case involved the husband assaulting the wife), and family (n = 10) or neighbors (n = 10). Notably, all injuries perpetrated by armed groups and Zaraguinas (n = 7) occurred in the north. Spouses accounted for 20-25% of incidents reported within each stratum.

Among children, whose ages ranged from 7 to 16 years (median = 11), 6 incidents involved fights, often with knives and mostly with other children. Exceptions included a 7-year-old in the north, who broke his arm while fleeing an armed group, and a 15-year-old girl in the south, hospitalized and with broken teeth suffered during a gang rape.

### Problems Facing Women and Children

At the beginning and end of interviews, respondents were asked general questions about problems facing women and children in their village or neighborhood. To analyze these responses, one researcher applied descriptive codes (using Excel) as they emerged from the data, and these codes were agreed-upon by the other members of the research team. In the northern stratum, the three most frequently raised issues related to health and health care (mentioned 176 times), food and nutrition (101), and water and sanitation (90). In the southern stratum, the most frequently mentioned areas of concern were health and health care (204), water and sanitation (109), and access to/quality of schooling (67), which was followed closely by support for agriculture and technology (including livelihood and income-generating activities) (66).

At the end of the interview, respondents were asked about what would help women cope with the problems facing them and their children in their village or neighborhood. In the northern stratum, the three most frequent responses related to support for health and health care (287), food and nutrition (182), and water and sanitation (157). In the southern stratum, the most frequently mentioned areas for support were health and health care (332), water and sanitation (192), and circulation of money (including support of microfinance projects and increased purchasing power) (132).

### Ethical Checks

Six clusters were revisited several days after the initial interviews took place, to assess whether respondents had experienced any adverse consequences (such as anger or resentment from neighbors, abuse from a family member or spouse) as a result of speaking with the research team. Interviewers were able to speak with 33 of the respondents they had previously interviewed, none of whom reported adverse consequences. Many women reported being happy to have participated in the interview process and felt that the survey was good for their country, and the husbands of several respondents reported feeling proud when their wives told them they had represented their family and their village in the survey. Three women asked when development projects would be brought to their villages, and one woman expressed survey fatigue.

## Discussion

Considerable barriers exist in using population-based surveys to capture data on conflict-related violations throughout an entire country. Nevertheless, and despite reports of grave violations in only a small fraction of households, evidence suggests a significant difference between the experiences of both children and adults living in the northern areas of CAR compared to those in the south. The majority of incidents involved adults over the age of 18, with rates of abduction, rape, killing and intentional injury significantly higher among adults. This evidence points to the need for strengthened protection programs not only for children, but for entire communities.

Armed actors (armed groups, Zaraguina, or government forces) were reported as perpetrators in approximately half of all incidents of killing, intentional injury, sexual abuse, rape, abduction, and forced recruitment among 5-17 year olds (55.8%) and persons 18 years or older (52.0%). While rape was the most frequently reported violation, three-quarters of all reported incidents were perpetrated by non-armed actors (such as husbands, neighbors, and strangers), indicating that the need for protection against sexual violence extends beyond areas of conflict. Conversely, abduction was the second most commonly reported violation and almost exclusively perpetrated by conflict actors and Zaraguinas. While humanitarian response in northern CAR is limited, programs intent on responding to protection issues such as gender-based violence and the recruitment and use of children in armed groups do exist. The high rate of abduction among both adults and children is evidence on which the humanitarian community should extend services. The large number of violations reportedly perpetrated by Zaraguinas also highlights the need for security actors to mitigate harm inflicted on civilians by armed, non-state actors acting outside of traditional organized groups.

Overall, the minority of reported violations occurred among children. Among these, only incidents perpetrated by armed combatants can be classified as falling within the purview of Resolution 1612 and therefore reportable to the UN Security Council. In 2008, the UN documented 58 such incidents [14], using the current case-based reporting system. If instead one uses the total number of violations by armed actors reported in this survey (Table 5), an estimate of the total number of grave violations likely to have occurred in the north and south of the country can be obtained.

**Table 5:** Reported Incidents of Grave Violations Against Children Perpetrated by Armed Actors

	ARMED GROUPS	ZARAGUINAS	CENTRAL AFRICAN MILITARY FORCES	TOTAL
**RECRUITMENT**				

*North*	8	2	0	**10**

*South*	0	0	0	**0**

**ABDUCTION**				

*North*	12	9	0	**21**

*South*	0	2	0	**2**

**RAPE**				

*North*	16	2	0	**18**

*South*	1	1	0	**2**

**SEXUAL ABUSE**				

*North*	8	1	0	**9**

*South*	0	0	0	**0**

KILLING*				

*North*	0	0	0	**0**

*South*	0	0	0	**0**

INTENTIONAL INJURY*				

*North*	1	0	0	**1**

*South*	0	0	0	**0**

**TOTALS**	**46**	**17**	**0**	**63**

* Killing and intentional injury are mutually exclusive categories.

In the northern stratum, 59 grave violations perpetrated by armed combatants were experienced in a sample of 2,461 children (aged 5-17 years) between January 2008 and July 2009. This suggests that approximately 8,180 violations (95% CI: 4,500-11,800) against children occurred in the northern areas of the country each year during the recall period. The discrepancy between the 58 cases reported to the UN Security Council that were recorded in 2008 and the estimate drawn from this sample point to the vast difference between the two monitoring approaches. This highlights the intrinsic value of a complementary system that would allow for population-based monitoring of trends over time alongside a verified case-based reporting system, essential for negotiating with armed groups and armed forces, and for fulfilling the UN Secretary General's reporting needs.

As discussed above, several potential biases likely affect this survey's results. If the sampling frame used to identify clusters was not complete, or if some groups were systematically not included in the sampling frame-such as those living outside of the country in 2003-and experienced violations at a rate different than the rest of the population, this could result in estimates being biased either toward or away from the null. Since security and logistical constraints prevented the survey team from reaching 9 of the 69 selected clusters, survey results are also affected if those living in the clusters not reached experienced violations more or less frequently over the course of the recall period, as compared to those living in accessible areas. Given that excluded clusters were often in volatile security environments, it is thus likely that their inclusion would have increased the rates estimated here. The same can be said for clusters that were not reachable and substituted by the next nearest village or neighborhood.

In situations where a group of 4 households was skipped due to no available respondents, bias could have occurred if the experience of those households was systematically different than that of the households interviewed. In one cluster where several groups of 4 households were skipped on the day of surveying, interviewers returned a day later and conducted interviews with those previously absent. Data from these interviews suggests skipped households experienced similar rates of violations as those interviewed on the previous day. However, the small number of households revisited in this way (11) makes this finding inconclusive. In 32% of households, no respondent was available at the initially selected household, but a respondent was located within one of the three neighbor households. If neighbors can report successfully on their neighbors, then this 32% of households not at home creates no potential bias. However, a potential source of bias could exist if neighbors consistently report incompletely about other neighbors and those not at home differed from their neighbors.

Interviewer performance was closely monitored through the first several clusters, to take note of any obvious differences in the number and types of violations reported. However, time constraints, logistics and a difference in interviewers' language abilities required the research team to occasionally divide the interviewers into separate teams and work in different locations with different security situations on different days. Because the violations did not occur evenly across the population, the areas where each interviewer conducted the most interviews, which differed for all interviewers, greatly affects the number of violations each interviewer recorded. Therefore, an accurate assessment of inter-rater reliability is difficult to obtain.

As with any survey, results are affected by respondents' knowledge, willingness to report, and ability to recall which events truly happened within the period under study. The neighborhood method also assumes respondents have the ability to report on events within their own households and in all neighboring households with equal consistency and that any hostile or difficult relationships between households or household members would not bias accurate reporting. While it is always difficult to verify the integrity of information recorded through surveys, the lack of a significant difference between the rates found through self-reports and neighbor-reports (Table 2) indicates that we can make the assumption that neighbors can report on their neighbors. The similar prevalence in both strata between the two kinds of reports diminishes concern for gross under-reporting of violations in neighbor households. The notable exception is rape, in which estimates derived from neighbor-reports differ markedly from those reported among respondents' own households.

Rape is an extremely sensitive topic, and difficulties in documenting its prevalence arise repeatedly in studies from a variety of settings and contexts [22,23]. In spite of slow, respectful interviews between two women, evidence of underreporting can be seen in this survey as well, and is particularly likely among neighbor-reports. The difference between the rates of rape reported is likely to have been influenced by the under-reporting of marital rape. We suspect that respondents might be unaware of marital rape occurring in neighboring households. In this survey, among 80 recorded marital rapes, 49 were self-reports and 31 were neighbor reports. As noted by Swiss, the true scale of violations can be suggested using additional means, including medical data such as the number of pregnancies known to be the result of rape [22]. Among the 181 incidents of rape reported in our data set, nine pregnancies are known to have resulted. Assuming that 1% of unprotected acts of sexual intercourse lead to pregnancy [22], and that this applies equally to all age groups, this would indicate a five-fold underreporting of rape within this sample.

Despite these difficulties, the survey instrument was sensitive enough to capture data that could be used to estimate the rate of most of the violations under study, and was able to detect a hundred times more incidents than those documented by the case-based MRM process. The use of a semi-structured, open-ended questionnaire is believed to be a main factor in allowing respondents to discuss difficult topics, including rape and sexual abuse. Clear differences in the rate of all violations for persons over 5 years of age (Table 3) indicate that the delineation between the northern and southern strata was a useful one that allowed for increased precision of the estimates obtained. The close supervision provided to the survey team also served to minimize the effect of interviewer bias on results, as all respondent and neighboring households were identified by a team leader. Interviewers reported that the women surveyed responded favorably to the use of buttons and bells to represent household members, and that it greatly assisted them in clarifying household composition as well as maintaining confidentiality.

The data provided through this exercise goes some way to building a base of comparison for future surveys and surveillance data from CAR. The method, which gleans information about four households through one interview, proved efficient in terms of both cost and time. The team was able to visit clusters in a large swath of the country while working under restrictive security constraints. At a cost of less than $50,000, it was possible to collect data despite limited funding, vehicles and personnel to do the work.

The emphasis on a need for health care, food, and water, identified through respondents' answers to two open-ended questions, points to the dire situation most Central Africans find themselves in, with even the most basic needs going unmet. Concerning health, many women spoke of the long distance to clinics and hospitals; the lack of qualified staff, equipment, and medicines once there; and the specific health needs of women and children. It is important to note that respondents were not asked to rank their needs and concerns, nor were they prompted to talk about more sensitive or personal topics. In addition to the most frequently mentioned areas, women also expressed needs and concerns related to insecurity; theft; lack of access to markets; transport and degraded roads and bridges; gender-based violence; support for vulnerable groups such as orphans and widows; lack of non-food items such as clothes and materials to build shelters; electricity; unemployment; single motherhood; difficulties cultivating due to rainfall, hazards from elephants, and attacks by armed groups; exploitation of natural resources and poaching; and the need for peace and reconciliation in CAR.

## Conclusions

This experience shows it is possible to collect representative, population-based estimates regarding the occurrence of grave violations in difficult and politically unstable settings. However, it is exceedingly difficult to calibrate innovative approaches to measuring grave violations when no gold standards exist. While it is likely that all events suffer from under-reporting, there is evidence that these events are occurring, particularly in the north of the country. Furthermore, respondents' articulation of the problems they and their communities face suggests that the dire health situation throughout the country cannot be ignored.

The neighborhood method was employed to allow respondents two different contexts in which they could discuss sensitive events-either within their households or within neighboring households-and as a means of accessing a large sample while working with limited time and resources. While this method may be sensitive enough to detect incidents, rates of violations in CAR may be too low to be detected precisely with a household survey. Nonetheless, these population-based figures greatly augment those presently available from case-based reporting systems. This survey and other recent, population-based work provide evidence that a security crisis is underway in CAR [24]. Yet establishing conditions on the ground is only one step in the accountability process. Both the national government and international actors present in CAR must acknowledge these dire conditions and actively work to better monitor and respond to them. Specifically, the Central African government, donors, international organizations such as the United Nations, and humanitarian aid organizations should make use of these data to better inform prevention and response programs, to assist in fundraising and advocacy, and to develop surveillance activities, which could provide ongoing, community-based monitoring of vital health and protection concerns.

## Competing interests

The authors declare that they have no competing interests.

## Authors' contributions

AP and KM participated in research design, data collection, analysis and drafting of the manuscript. LR supervised the research design, data collection and analysis. All authors read and approved the final manuscript.
